# Bilateral Total Hip Prosthesis in Coxarthritis of Inflammatory Origin: Technical Features and Intraoperative Complications Encountered in Five Cases

**DOI:** 10.7759/cureus.52242

**Published:** 2024-01-14

**Authors:** Samir Ben Salah, Ayman Ben Abdellah, Adnane Lachkar, Hicham Yacoubi, Najib Abdeljaouad

**Affiliations:** 1 Orthopedic Trauma Department, Mohammed VI University Hospital, Faculty of Medicine and Pharmacy of Oujda, Mohamed I University, Oujda, MAR

**Keywords:** intraoperative complications, technical features, coxarthritis, inflammatory origin, bilateral total hip prosthesis

## Abstract

Total hip arthroplasty remains the treatment of last resort in inflammatory coxarthritis, where joint destruction is bilateral as well as the treatment which must be bilateral. We present in this work the experience of our orthopaedic department with a series of five cases (10 hips) operated for total hip arthroplasty. the first particularity observed in this series is the abnormal bone fragility present in 55% of the cases. For this reason, we had two other intraoperative complications that are related to this bone fragility, during the preparation of the acetabulum, we had a destruction of the medial wall by the burr that went unnoticed intraoperatively and was discovered during the patient's recovery from acute ischemia secondary to the burr, which led to an extensive rupture of the common femoral vein and partial sectioning of the common femoral artery.

We also had an exceptional incident in a case with two ankylosed hips; in fact, when the approach was performed, the sciatic nerve was found pressed against the posterior surface of the greater trochanter, which was unusual but was explained by the retraction of the structures of the gluteal region secondary to prolonged immobilization. Thus, there was one case of cement shock manifested by hypotension occurring immediately after cement placement. In front of this inflammatory disease and ankylosis terrain, the surgeon must always be prepared for any complication and must keep in mind that he is operating on a hip that is anatomically not normal due to ankylosis and retraction of the vascular, nerve, and muscle structures.

## Introduction

Coxarthritis secondary to inflammation is dominated by inflammatory spondyloarthritis in the first place, followed by rheumatoid arthritis. The term spondyloarthritis refers to a group of immune-mediated diseases characterized by inflammation of the axial skeleton, peripheral joints, and enthesis. Ankylosing spondylitis is the most common and characteristic of these entities, and our understanding of the underlying mechanism of the disease remains incomplete [1,2].

At the hip level, surgery remains the almost imperative recourse for all patients with coxarthritis secondary to ankylosing spondylitis or rheumatoid arthritis in the absence of a medical treatment that allows a definitive cure of the inflammatory diseases and consequently stopping the destructive process of the hip secondary to the inflammation [3-5].

## Case presentation

Material and methods

This work presents the experience of our trauma and orthopedics department with 10 hips (five patients diagnosed and followed in the rheumatology department for inflammatory disease) collated over an eight-year period from January 2015 to January 2023. All patients were admitted for the management of coxarthritis at the surgical stage secondary to ankylosing spondylitis in five cases. 

All patients underwent total hip replacement (dual mobility prosthesis with a metal-polyethylene friction couple) initially on the most symptomatic and disabling side and then on the other side after six months. A front-view hip X-ray checked the location of the prosthesis in all patients immediately postoperatively. All patients were followed up on consultation every three months for one year, and every six months with a clinical hip examination and a standard hip radiograph.

The collection of data of all patients is done through our computerized system of archiving medical records, which allows easy access at any time to medical data of patients and their evolution.

Case presentation

Case 1

**Figure 1:**
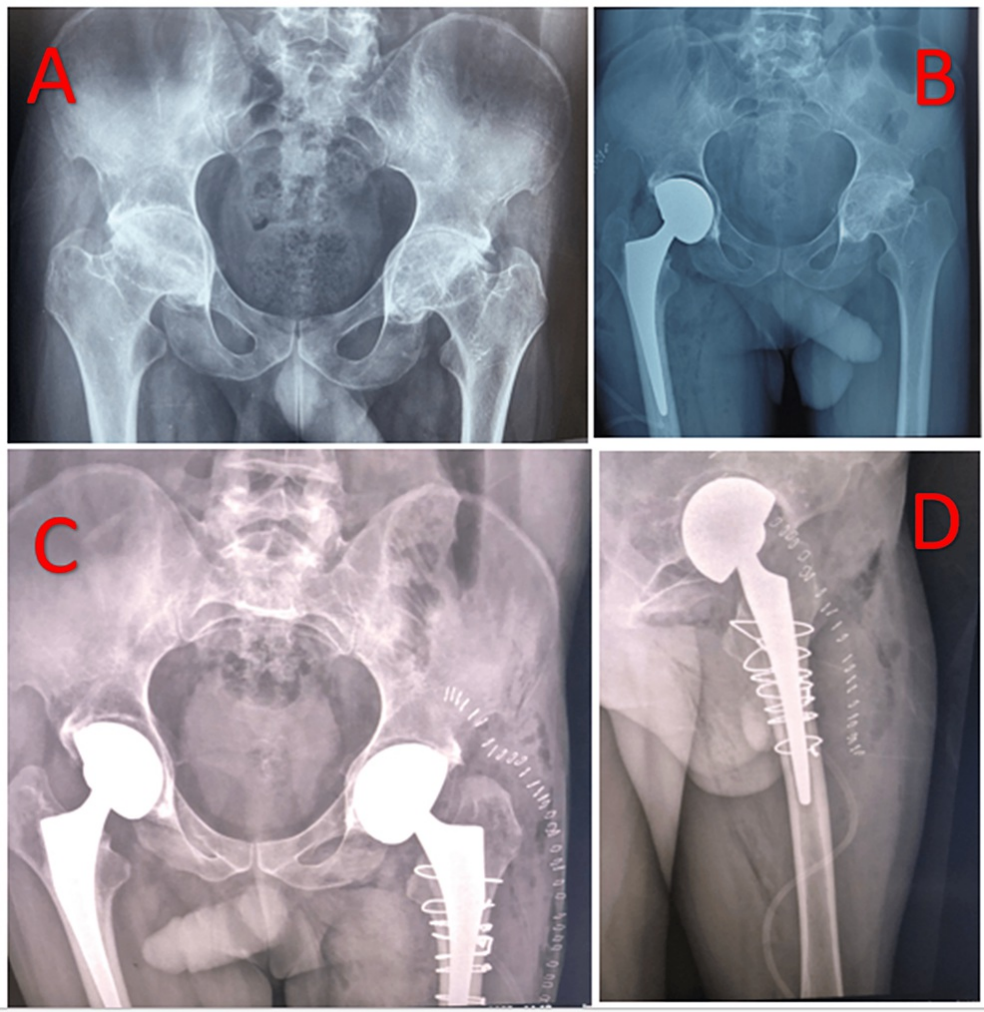
Radiographic assessment of case 1 A: X-ray of the pelvis showing bilateral coxarthritis at the ankylosis stage. B: Control radiograph after the right prosthesis had been fitted. C: pelvis X-ray after completion of the left prosthesis, complicated intraoperatively by a peri-prosthetic fracture treated by steel wire cerclage. D: Immediate postoperative hip X-ray, profile view.

This is the case of a 40-year-old patient, diagnosed and followed for 15 years with ankylosing spondylitis. The patient was admitted for management of secondary coxarthritis at the stage of bilateral ankylosis. The patient was operated on initially for a total right hip prosthesis, with no complications apart from unusual bone fragility. The patient resumed treatment after six months for management of the left hip and had a periprosthetic fracture during the reduction of the definitive prosthesis as a complication. Our approach to this complication was to widen the posterolateral approach from Moore downwards, in line with the femur, until the fracture was exposed. We reduced the fracture with two reduction forceps, stabilized the reduction with steel wires, and finally fitted a cemented stem (Figure 1).

Technical note: In the event of a periprosthetic fracture occurring intraoperatively, one must always begin by reducing and osteosynthesizing the fracture, as the cement will become interposed in the fracture sites, preventing reduction and giving the fracture a high potential for pseudarthrosis.

Case 2

**Figure 2:**
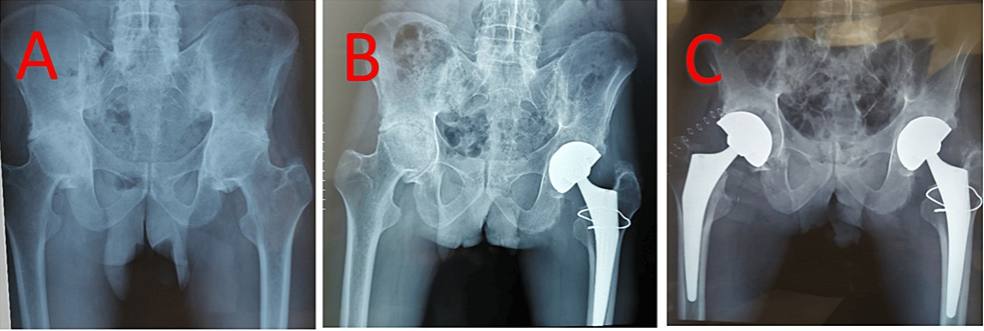
Radiographic assessment of case 2 A: Initial radiograph. B: Follow-up radiograph after placement of the left prosthesis. C: Follow-up radiograph after fitting the right prosthesis.

This is the case of a 36-year-old man who had been treated for ankylosing spondylitis for 10 years. Initially operated on the left hip, the complication was a peri-prosthetic fracture that occurred during the preparation of the shaft. The fracture was simple and accessible, stabilized by a steel wire. Returned after eight months for management of the right hip, which passed without complication apart from abnormal bone fragility (Figure 2).

Case 3

**Figure 3:**
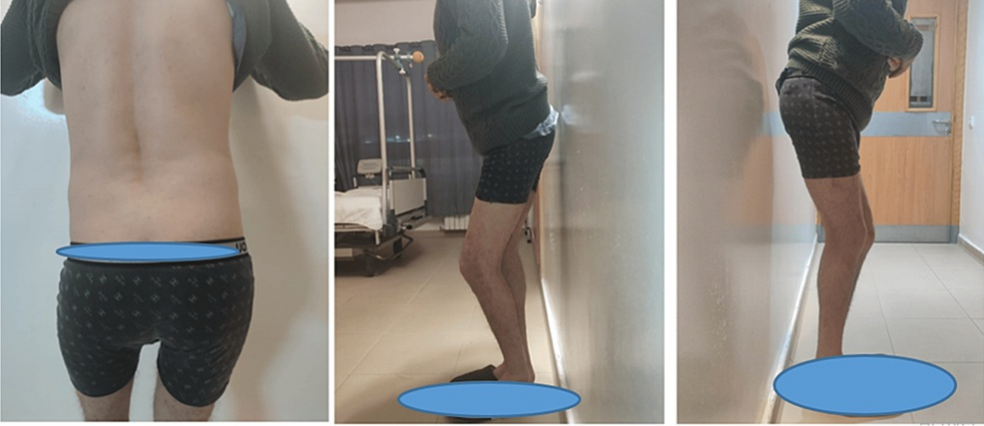
Clinical aspect of case 3 with bilateral ankylosis who remained immobile for a long time

**Figure 4:**
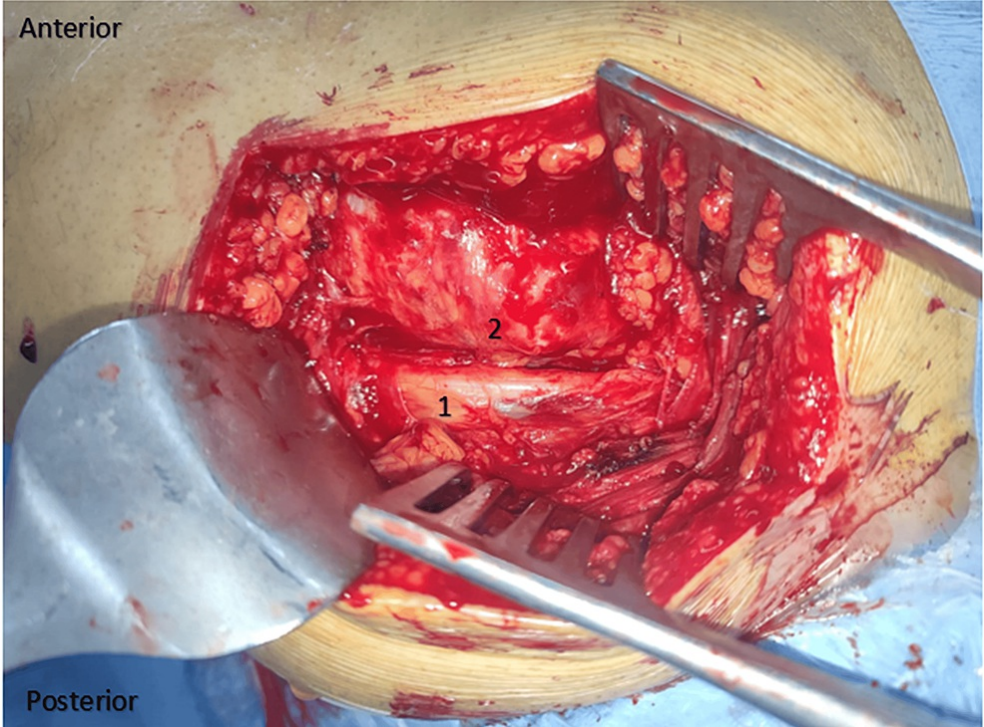
Intraoperative image of case 3 The image is showing an unusual location of the greater sciatic nerve glued to the posterior aspect of the greater trochanter. 1: Posterior aspect of the greater trochanter. 2: Greater sciatic nerve.

**Figure 5:**
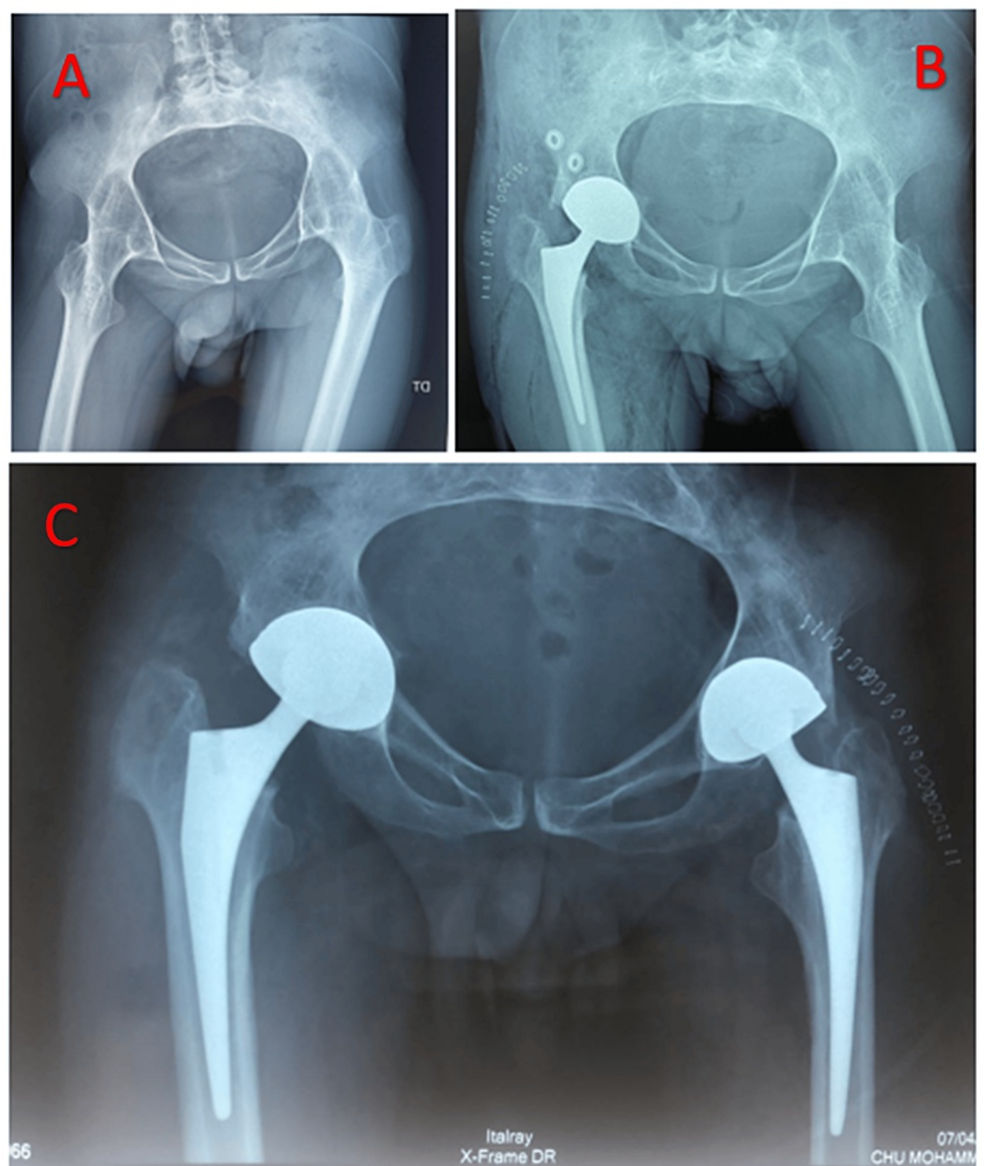
Radiographic findings in the case 3 patient with an abnormal location of the sciatic nerve A: Front pelvis X-ray showing bilateral ankylosis of both hips secondary to ankylosing spondylitis. B: Postoperative control pelvis radiograph after the first prosthesis on the right, which passed without complication. C: Postoperative control pelvis radiograph after the second prosthesis on the left, during which this anatomical peculiarity was revealed.

We had this experience with a single case of a 42-year-old gentleman who had been treated for ankylosing spondylitis for 18 years, and in whom the coxarthritis was at the ankylosis stage. When the prosthesis was performed on the left side (note that we didn't have this problem when the first prosthesis was performed on the right), we faced a sciatic nerve stuck on the posterior surface of the greater trochanter. Indeed, given this particular and abnormal location of the greater sciatic nerve, which was attached to the posterior surface of the greater trochanter, we were obliged to carefully dissect around the nerve in order to isolate and protect it. This patient also had very fragile pelvic trochanters, and we couldn't even put them on a traction wire to recline them backward in order to protect the nerve. So we had to work alongside the nerve, protecting it with lakes throughout the operation. Also, given the position of the nerve on the greater trochanter, we were obliged to cut the pelvic trochanters almost flush with the bone, which led to considerable bleeding that we were unable to control, resulting in a state of hemorrhagic shock stabilized by drugs and transfusion (Figures 3-5).

Case 4

**Figure 6:**
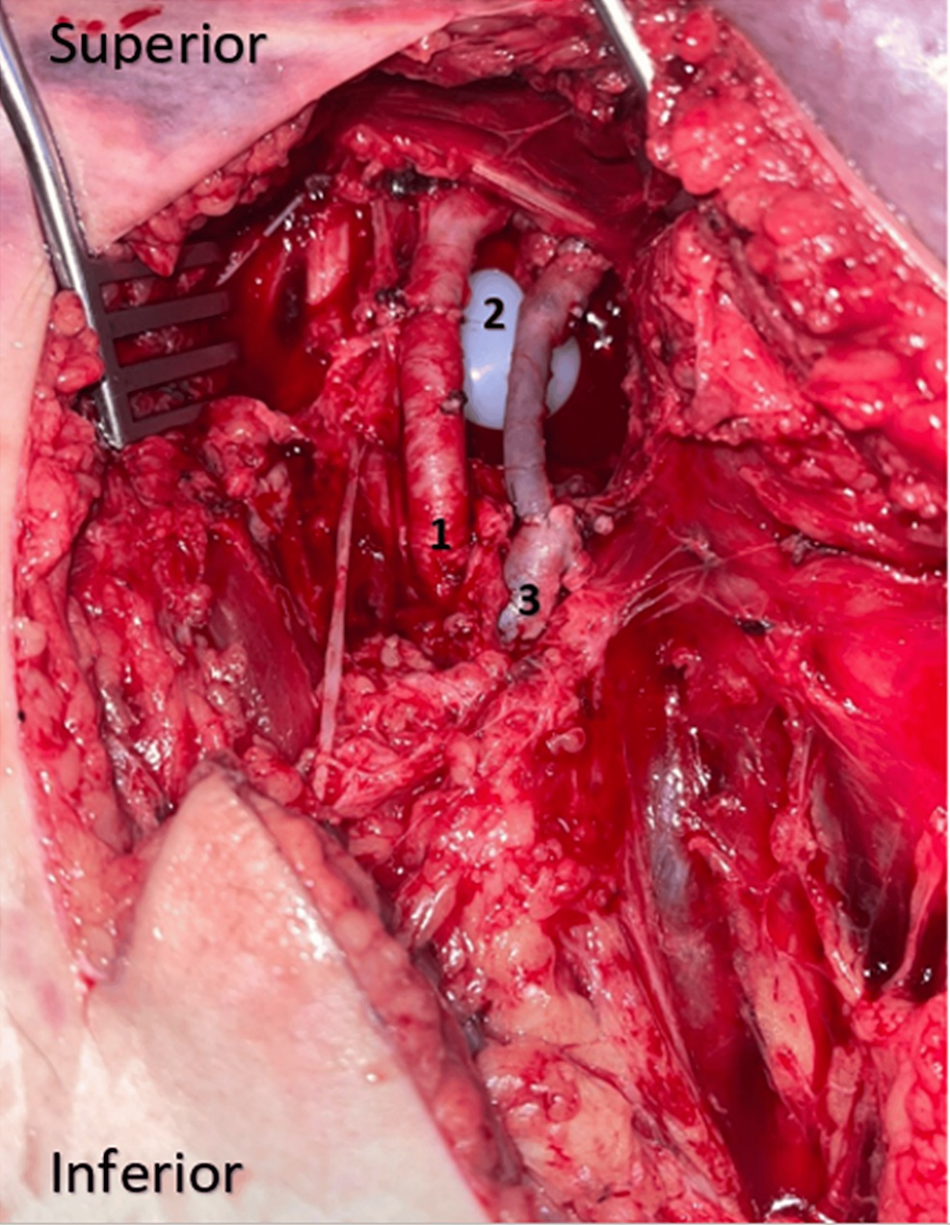
Intraoperative image of the patient in case 4 with vascular complication 1: Common femoral artery after suturing. 2: Polyethylene prosthesis visible intra-pelvic after the destruction of the medial wall by the milling effect. 3: Common femoral vein after bypass surgery.

In all patients, unusual bone fragility was observed, which is why we tried to go slowly and carefully, but despite this, we had a vascular complication during the preparation of the acetabulum caused by milling in a 45-year-old patient being treated for ankylosing spondylitis. The fragility of the coxal bone was observed from the outset, and the acetabulum was prepared by progressive milling down to size 50, followed by the fitting of an uncemented size 50 definitive acetabulum. Postoperatively, the patient presented with acute ischemia of the operated lower limb, for which reason she underwent an angioscan showing thrombosis of the common femoral vein. The patient was therefore taken back to the operating room by the vascular surgery team in the evening via an anterior approach to the femoral pedicle. Surgical exploration revealed an extensive complete rupture of the common femoral vein, requiring grafting and partial sectioning of the common femoral artery (Figure 6).

Case 5

**Figure 7:**
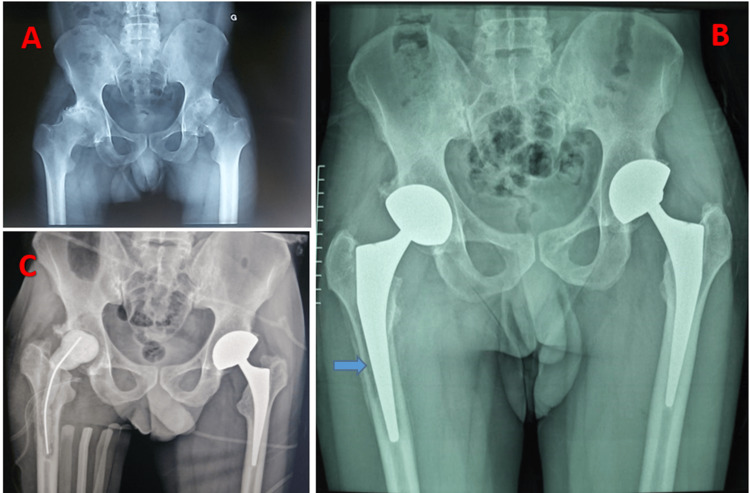
Radiographic findings in the patient in case 5 with biologic cement reaction shock A: Front pelvis radiograph showing bilateral coxarthritis at the ankylosis stage. B: Radiograph of the front pelvis showing radiographic signs (blue arrow) of septic loosening. C: Post-operative control pelvis radiograph after placement of biologic cement.

This is a case of rheumatoid arthritis treated with bilateral hip arthroplasty, with a septic complication on the right prosthesis after three months. This involved a revision (removal of the infected prosthesis and insertion of a cement spacer). The patient was admitted to the operating theatre under locoregional anaesthesia; the old Moore approach was used, with pus coming out at the front during arthrotomy, and the prosthesis was removed with abundant saline lavage. When the spacer was inserted, the patient went into shock with cardiac arrest and was resuscitated in the operating room. The patient died after 48 hours in the intensive care unit (Figure 7).

## Discussion

An osteoarthritic hip at the ankylosis stage, in most cases secondary to ankylosing spondylitis, poses real technical problems during management, initially during preoperative planning due to the absence of the non-pathological contralateral side, which facilitates and guides planning [4,5]. The same applies to patients with end-stage ankylosing spondylitis presenting a pelvic rotation disorder compensated by hyperextension of the hip [6,7]. Therefore, given that joint destruction and ankylosis, especially in inflammatory diseases, are bilateral, treatment can only be bilateral [8,9]. The first technical problem with a patient with two ankylosed hips arises as soon as the patient is installed, which is very difficult on an orthopedic table [8-10]. This is a minor challenge for teams used to the orthopedic table and the anterior approach, but installation in lateral decubitus on an ordinary table seems to solve the problem [6-11].

An ankylosed hip equals a hip that has been immobile for a certain length of time, which means that a retracted gluteal region may be the cause of malposition of the vasculonervous structures (the greater sciatic nerve and the superior gluteal pedicle) passing through this region, which must not be damaged during surgery [5,10,12]. This was the case in a patent included in our series, where the greater sciatic nerve was found pressed against the posterior aspect of the greater trochanter. In this context, an indication for preoperative MRI may even be discussed in patients with ankylosed hips who have been immobile for a very long time, with clinically evident amyotrophy of the gluteal region [11-14]. In whom there is very likely to be a retraction of the anatomical structures of this region or even malposition of the greater sciatic nerve or the superior gluteal pedicle, so if this feature is identified preoperatively on MRI data, surgeons can easily avoid a high risk of intraoperative nerve damage. The nerve can be isolated and protected straightaway, alternatively, even if possible, the posterolateral approach can be changed for another that doesn't pass through the posterior region (the transtrochanteric external approach remains the preferred approach in these cases, as it gives very good exposure with less risk apart from the greater trochanter osteotomy) [5,10,12,14].

In addition, on an immobile hip, the pelvitrochanteric muscles and gluteus medius are very fragile and can be the site of fatty infiltration, especially on a hip that has remained immobile for a long time. Therefore, we need to think very early about the stability of the prosthesis before it is performed, and implement a good protocol for progressive rehabilitation of the gluteus medius postoperatively [8].

At the femoral section stage, the femoral neck must first be exposed medially until it fuses with the hip bone, and laterally until it fuses with the greater trochanter [3,7]. During this phase of the operation, periarticular ossifications, if present, should always be carefully resected using gauge forceps or bone chisels. In our series, none of the cases had periarticular ossifications, and we always made a first subcapital femoral cut (using a straight bone chisel), taking the upper edge of the obturator frame as a reference, to be able to free the joint. When making the first femoral cut, we can quantify the degree of bone fragility, because when preparing the acetabulum, we gradually and gently cut into the femoral head with a burr, taking care not to destroy the medial wall. In all cases, a second femoral cut was made 1 cm from the lesser trochanter, and the femoral preparation was performed progressively to a size allowing good primary stability. In our series, and during femoral preparation, we had a periprosthetic femur fracture in three cases, due to bone fragility in this type of patient.

Prosthetic surgery on inflammatory hips should not be standardized; it includes these particularities, as well as increased risks of certain complications compared to non-inflammatory hips [6-8,12]. We're working on a very fragile bone. So, we needed to be careful when handling this fragile bone, both when preparing the acetabulum, to avoid destroying the medial wall and when preparing the femur to avoid generating periprosthetic fractures, which will prolong the operating time and increase the risk of infection, which will have an impact on the postoperative course and the final outcome of the surgery [4,5,8,11]. In the case of hips that have been ankylosed for a long time, it is important to take into account the retraction of structures in the gluteal region, which can lead to unexpected localization of the sciatic nerve and the superior gluteal pedicle, which can be accidentally injured intraoperatively. The small number of patients included in this series limited our study [2,5,6,9,13].

## Conclusions

This little experience opened our minds to the particularities of patients with inflammatory coxarthritis, which must be taken into account in all hip prosthetic surgery, as they can be serious intraoperative complications that are difficult to manage. In particular, the unusual bone fragility present in the majority of patients with inflammatory coxarthritis increases the risk of fracture during femoral and acetabular preparation. Thus, we must be wary of patients with ankylosed hips who remain immobile for long periods, due to retraction of the soft tissues in the gluteal region, which can be the source of unusual localization of the sciatic nerve, which can be accidentally injured during the posterior approach.
